# Birt-Hogg-Dubé syndrome with novel FLCN gene mutations and different clinical presentations: Case series

**DOI:** 10.5578/tt.202401844

**Published:** 2024-03-26

**Authors:** Ayşegül ERİNÇ, Damla AZAKLI, Gülsüm KIRHAN, Celal SATICI

**Affiliations:** 1 Clinic of Pulmonology, Yedikule Chest Disease and Thoracic Surgery Training and Research Hospital, İstanbul, Türkiye

## Abstract

**ABSTRACT**

**
Birt-Hogg-Dubé syndrome with novel FLCN gene mutations and different clinical presentations: Case series
**

*
Birt-Hogg-Dubé syndrome (BHDS) is a rare genetically inherited autosomal dominant condition characterized by pulmonary cysts, spontaneous pneu- mothorax, benign skin tumors (fibrofolliculoma, trichodiscoma), and renal tumors. Birt et al. defined this genodermatosis as being caused by numerous germline mutations in the FLCN gene, which encodes the protein folliculin located in the 14th exon of the p11.2 region of the 17th chromosome. Although this rare syndrome should be kept in mind in patients with cystic lung disease, it can also be seen without lung involvement. Furthermore, with an increasing number of reported cases, certain mutations have been cor- related with specific clinical manifestations, enabling tailored follow-up pro- tocols based on the type of genetic mutation.
*

**Key words:** *Cystic lung disease; genodermatosis; rare disease
*

**ÖZ**

**
Yeni FLCN gen mutasyonu ve farklı klinik prezentasyonlarla Birt Hogg Dubé sendromu: Olgu serisi
**

*
Birt Hogg Dubé sendromu (BHDS), pulmoner kistler, spontan pnömotoraks, iyi huylu deri tümörleri (fibrofoliküloma, trikodiskoma) ve böbrek tümörleri ile karakterize nadir görülen otozomal dominant geçişli bir hastalıktır. Birt ve arkadaşları bu genodermatozu, 17. kromozomun p11.2 bölgesinin 14. ekzo- nunda bulunan folliculin’i kodlayan FLCN genindeki çok sayıda germline mutasyonun neden olduğu bir hastalık olarak tanımlamıştır. Bu nadir send- rom, kistik akciğer hastalığı olan hastalarda akılda tutulması gerekse de akci- ğer tutulumu olmadan da görülebilir. Ek olarak, daha fazla vaka bildirildikçe, bazı mutasyonlar klinik belirtilerle ilişkilendirilmeye başlanmıştır ve takip protokolleri genetik mutasyon tipine göre bireyselleştirilebilir.
*

**Anahtar kelimeler:** *Kistik akciğer hastalığı; genodermatoz; nadir hastalık
*

## INTRODUCTION

Birt-Hogg-Dubé syndrome (BHDS) is a rare geneti- cally inherited autosomal dominant condition char- acterized by pulmonary cysts, spontaneous pneumo- thorax, benign skin tumors (fibrofolliculoma, tricho- discoma), and renal tumors (1). Birt et al. defined this genodermatosis as being caused by numerous ger- mline mutations in the FLCN gene, which encodes the protein folliculin located in the 14th exon of the p11.2 region of the 17th chromosome (2,3). The pres- ence of at least one of the major criteria and at least two of the minor criteria are needed to diagnose BHDS. The major criteria were defined as having more than five fibrofolliculoma or trichodiscoma lesions and FLCN gene mutations in adulthood, with at least one histopathologically confirmed. Minor criteria were defined as cystic lung disease on tho- racic imaging, a history of kidney tumor, and a first- degree relative with BHDS (4). Although this rare syndrome should be kept in mind in patients with cystic lung disease, it can also be seen without lung involvement (5). Furthermore, with an increasing number of reported cases, certain mutations have been correlated with specific clinical manifestations, enabling tailored follow-up protocols based on the type of genetic mutation (6).

In accordance with this, our aim is to present three BHDS patients with distinct FLCN gene mutations, each exhibiting unique clinical presentations. We believe these cases merit presentation due to their unique characteristics: the first patient harbored a novel FLCN gene mutation previously unreported, the second patient exhibited a mutation reported only once thus far, and the third patient presented with the most frequent FLCN gene mutation, showcasing diverse clinical manifestations.

## CASE REPORT

**CASE 1: A 66-year-old male patient**

A patient with a history of spontaneous pneumothorax applied to us for a follow-up two years ago. The thoracic computed tomography (CT) scan of the patient revealed perifissural, paracardiac, bibasilar, and elliptic/lentiform-shaped cysts with vascular structures on their walls (Figure 1). The patient, who was an active smoker, was scheduled for further evaluation to assess for cystic lung diseases. The Anti- SSA/Anti-SSB testing, requested for the differential diagnosis of LIP to exclude a potential association with Sjögren’s syndrome, returned negative results.

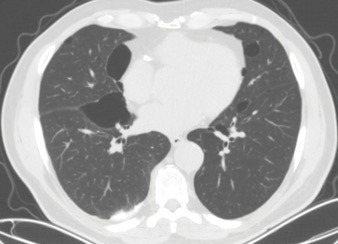

**Figure 1.** An axial section of lung window of chest CT image showing bilateral multiple lung cysts (1st patient).

For BHDS, abdominal ultrasonography (USG) for renal pathologies was requested: bilateral renal multiple peripelvic and cortical cysts, hyperechoic lesion in the left renal parenchyma were observed, and yearly follow-up was planned in terms of angiomyolipoma/complicated cyst differentiation. FLCN gene mutation was reported as the “c.671_672del’ mutation” which was extremely rare (according to the Leiden Open Variation Database ( https://www.lovd.nl/ ) (Table 1). Only one case with this mutation was reported; however, no data regarding clinical presentation was available (7). The asymptomatic patient with no skin involvement was scheduled for annual follow-up assessments focusing on renal pathologies.

## CASE 2: A 43-year-old male patient

The patient, an active smoker with a history of pneumothorax, presented to our clinic with complaints of cough and shortness of breath persisting for a year. There were elliptical, non-uniformly confined bibasilar, paracardiac cysts in the thorax CT (Figure 2). The higher number and the larger size of the cysts were remarkable. S100 and CD1a were found to be negative in the bronchoalveolar lavage (BAL) conducted to rule out Histiocytosis X. Anti-SSA/ Anti-SSB testing returned negative results, prompting further investigations including FLCN gene mutation analyses and an abdominal ultrasound (Table 1). The patient was diagnosed with BHDS with a positive FLCN gene mutation “c.1326del mutation” without any renal pathology on abdominal USG. To our knowledge, this is the first documentation of a novel mutation of the FLCN gene in a patient with BHDS (according to the Leiden Open Variation Database

Erinç A, Azaklı D, Kırhan G, Satıcı C.

**Table:** 

**Table 1.** Folliculin (FLCN) gene mutations			
**Patients**	**Folliculin (FLCN) gene mutation**	** Clinical manifestation and organ involvement **	**Radiologic finding**
1st case	c.671_672del	Pneumothorax Pulmonary cysts Renal cysts	Perifissural, paracardiac, bibasilar, elliptic/lentiform shaped cysts with vascular structures on the wall
2nd case	c.1326del	Pneumothorax Pulmonary cysts	Elliptical, non-uniformly con- fined bibasilar, paracardiac cysts
3rd case	c.1285dupC	Pneumothorax Pulmonary cysts Fibrofolliculoma	High number of bilateral, non-uniformly circumscribed, perivascular cysts

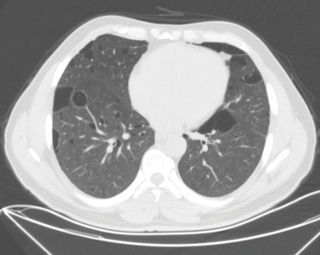

**Figure 2.** An axial section of lung window of chest CT image showing bilateral multiple lung cysts (2nd patient).

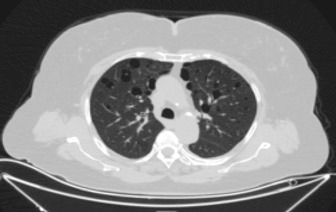

**Figure 3.** An axial section of lung window of chest CT image showing bilateral multiple lung cysts (3rd patient).

( https://www.lovd.nl/ ) (Table 1). The asymptomatic patient with no skin involvement was scheduled for annual follow-up assessments focusing on renal pathologies.

## CASE 3: A 66-year-old female patient

The patient, who had known hypertension, had never smoked, and had a history of pneumothorax, was admitted to our clinic for shortness of breath that persisted for two years. There was a significant number of bilateral, non-uniformly circumscribed, perivascular cysts localized in both upper and lower lobes (Figure 3). A workup test was planned for cystic lung disease. Skin lesions suggestive of BHDS were observed, prompting a referral to dermatology for further evaluation. The patient was diagnosed with BHDS and abdominal USG revealed no evidence of renal pathology (Table 1). A skin biopsy confirmed fibrofolliculoma. The asymptomatic patient with skin involvement was scheduled for annual follow-up assessments focusing on renal pathologies.

## CONCLUSION

Birt-Hogg-Dubé syndrome is a rare, inherited, autosomal dominant disorder first described in 1977. Initial cases were described in a case series involving similar patients with skin manifestations, including cutaneous fibrofolliculoma/tricodiscoma. According to recent literature, BHDS has been reported in approximately 200 families (8). Therefore, it should be considered in the diagnostic evaluation of cystic lung diseases. Although lung cysts are common, they are usually asymptomatic unless pneumothorax develops c.1285dupC was the most frequently reported FLCN gene mutation, as reported by Xiaowen Hu et al., with a frequency of 17.4% (5). In the same study, it was demonstrated that the c.1285dupC FLCN subtype increased the risk of developing pneumothorax. Although mutations can arise anywhere in the FLCN gene including exons

and introns, 50% of the reported mutations are frameshifts caused by insertions or deletions at the mutational hotspot in exon 11’s cytosine eight nucleotide (9,10). To present, no association between genotype and phenotype has been found, and additional research is required. However, some investigators have shown a higher prevalence of exon

11 mutations in patients with a history of pneumothorax, as well as a link between exon 9 and 12 mutations and the number and size of lung cysts, respectively (6). Other researchers have suggested that individuals with the c.1285delC mutation may have a lower risk of developing kidney cancer, although additional research is warranted (11).

In our case series, all of our patients with different mutation subtypes developed spontaneous pneumothorax. Further research on a larger population is needed to explore the correlation between these mutations and phenotype. FLCN genetic testing methods are extremely important in the diagnosis of BHD syndrome. To fully understand the association between FLCN mutation subtypes and disease phenotypes, mutation analysis must be performed in each patient. Renal tumors represent the most serious complication of the disease, emphasizing the importance of timely diagnosis and treatment to prevent the development of metastatic disease. Due to the increased risk of metastatic renal cancer, follow-up and screening is important. To date, no definitive treatment for BHDS has been reported. Early detection of renal tumors in BHDS patients is crucial for patient education and family counseling. All our patients are undergoing regular monitoring for potential renal tumor development, as it represents the leading cause of morbidity and mortality in BHDS.

## CONFLICT of INTEREST

The authors have no conflict of interest to declare.

## AUTHORSHIP CONTRIBUTIONS

Concept/Design: AE, CS Analysis/Interpretation: AE, CS Data acqusition: All of authors Writing: All of authors Clinical Revision: AE, CS

Final Approval: AE, CS

## REFERENCES

1. *
  Jensen DK, Villumsen A, Skytte AB, Madsen MG, Sommerlund M, Bendstrup E. Birt-Hogg-Dubé syndrome: A case report and a review of the literature. Eur Clin Respir J 2017; 4: 1292378. https://doi.org/10.1080/20018525.2 017.1292378*
2. *
  Nickerson ML, Warren MB, Toro JR, Matrosova V, Glenn G, Turner ML, et al. Mutations in a novel gene lead to kidney tumors, lung wall defects, and benign tumors of the hair follicle in patients with the Birt-Hogg-Dubé syndrome. Cancer Cell 2002; 2: 157-64. https://doi.org/10.1016/ S1535-6108(02)00104-6*
3. *
  Birt AR, Hogg GR, Dubé WJ. Hereditary multiple fibrofol- liculomas with trichodiscomas and acrochordons. Arch Dermatol 1977; 113(12): 1674-9. https://doi.org/10.1001/ archderm.1977.01640120042005*
4. *
  Schmidt LS, Linehan WM. Molecular genetics and clinical features of Birt-Hogg-Dubé syndrome. Nat Rev Urol 2015; 12: 558. https://doi.org/10.1038/nrurol.2015.206*
5. *
  Satıcı C, Köybaşı G, Koyukan A, Seyhan EC. A rare coexis- tence of Birt-Hogg-Dubé syndrome and sarcoidosis. Respir Case Rep 2021; 10(3): 186-9. https://doi.org/10.5505/ respircase.2021.40327*
6. *
  Toro JR, Pautler S, Stewart L, Glenn G, Weinreich M, Toure O, et al. Lung cysts, spontaneous pneumothorax, and genetic associations in 89 families with Birt-Hogg-Dubé syndrome. Am J Respir Crit Care Med 2007; 175: 1044-53. https://doi.org/10.1164/rccm.200610-1483OC*
7. *
  López V, Jordá E, Monteagudo C. Actualización en el sín- drome Birt-Hogg-Dubé [Birt-Hogg-Dubé syndrome: an update]. Actas Dermosifiliogr 2012; 103(3): 198-206. https://doi.org/10.1016/j.ad.2011.07.009*
8. *
  Menko FH, van Steensel MA, Giraud S, Friis-Hansen L, Richard S, Ungari S, et al. European BHD Consortium. Birt- Hogg-Dubé syndrome: Diagnosis and management. Lancet Oncol 2009; 10: 1199-206. https://doi. org/10.1016/S1470-2045(09)70188-3*
9. *
  Lim DH, Rehal PK, Nahorski MS, Macdonald F, Claessens T, Van Geel M, et al. A new locus-specific database (LSDB) for mutations in the folliculin (FLCN) gene. Hum Mutat 2010; 31: E1043-51.
  *
10. *
  Wei MH, Blake PW, Shevchenko J, Toro JR. The folliculin mutation database: An online database of mutations asso- ciated with Birt-Hogg-Dubé syndrome. Hum Mutat 2009; 20: E880-90. https://doi.org/10.1002/humu.21075*
11. *
  Toro JR, Wei MH, Glenn GM, Weinreich M, Toure O, Vocke C, et al. BHD mutations, clinical and molecular genetic investigations of Birt-Hogg-Dubé syndrome: A new series of 50 families and a review of published reports. J Med Genet 2008; 45: 321-31. https://doi.org/10.1136/ jmg.2007.054304*

